# Charge‐Acoustic Phonon Coupling Determined Carrier Transport Properties in FA_x_MA_1−x_PbBr_3_ Perovskite Single Crystals

**DOI:** 10.1002/advs.202524220

**Published:** 2026-02-13

**Authors:** Wei Zhang, Zheng Zou, Tiehui Wu, Zijie Xiao, Dan Feng, Jianbin Zhong, Xianshao Zou, Ningjiu Zhao

**Affiliations:** ^1^ School of Physics and Materials Science Guangzhou University Guangzhou China; ^2^ Max Planck Institute For Polymer Research Mainz Germany; ^3^ Songshan Lake Materials Laboratory Dongguan China; ^4^ Qingdao Innovation and Development Center Harbin Engineering University Qingdao China

**Keywords:** carrier mobility, charge transport, elastic modulus, perovskite single crystal

## Abstract

Mixed‐ion perovskites have attracted wide attention in optoelectronic applications due to their exceptional properties, such as tunable bandgaps and long carrier diffusion lengths. Carrier mobility is a critical factor for determining the performance of optoelectronic devices. However, the substantial variation in reported mobility values has hindered a comprehensive understanding of charge transport in mixed‐ion perovskites. In this work, we conducted time‐domain Brillouin scattering measurements to characterize the elastic constants and deformation potentials of electrons/holes in FA_x_MA_1−x_PbBr_3_ single crystals. We find that the elastic constants of crystals decrease with the increase of FA content. Applying deformation potential theory to these measured parameters, we determined the intrinsic carrier mobility determined by carrier‐acoustic phonon coupling, and revealed high intrinsic electron and hole mobilities in FA_x_MA_1−x_PbBr_3_ single crystals. We also find that both electron and hole mobilities, determined by carrier‐acoustic phonon coupling, decrease with the increase of FA content in FA_x_MA_1−x_PbBr_3_ single crystals. This work advances the comprehension of intrinsic carrier transport properties and offers essential guidance for the rational design of high‐performance perovskite materials.

## Graphical abstract

This study investigates acoustic phonon‑carrier coupling in FA_x_MA_1−x_PbBr_3_ single crystals using time‑domain Brillouin scattering. The results show that increasing the FA content in FA_x_MA_1−x_PbBr_3_ single crystal reduces both the elastic constant and the intrinsic carrier mobility governed by the acoustic phonon‑carrier coupling.

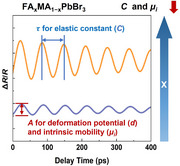

## 1 Introduction

Organic–inorganic halide perovskites (OIHPs) are promising candidates for optoelectronic devices, such as solar cells [1], light‐emitting diodes [2], and photodetectors [3], owing to their attractive properties, including a high absorption coefficient [4] and superior charge‐carrier mobility [5]. Among them, mixed‐cation FA_x_MA_1‐x_PbBr_3_ single crystals stand out as an important OIHP system, whose bandgap tunability further enhances their potential for light‐emitting diodes and solar cells [6, 7]. Since charge‑carrier mobility is a key determinant of device performance [8, 9], elucidating the fundamental mechanisms that govern mobility is essential for the further optimization of perovskite‑based devices.

The factors governing carrier mobility of perovskite can be mainly classified into two scattering mechanisms, extrinsic and intrinsic [10, 11, 12]. The extrinsic scattering mechanism can arise from defects, such as grain boundaries, energetic disorder, or impurities. While intrinsic scattering mechanism originates from the interaction of charge carriers with the lattice and depends on the fundamental properties of the material. To detect the charge transport in OIHPs, a variety of techniques have been employed to measure carrier mobility in OIHPs, which fall into two main categories: contact‐based electrical methods and contact‐free optical methods [13]. Electrical approaches include time‐of‐flight (TOF) [14], space charge limiting current (SCLC) [15], and Hall‐effect measurements [16]. Optical techniques encompass terahertz time‐domain spectroscopy (THz‐TDS) in both steady‐state and pump‐probe configurations, as well as transient absorption (TA) spectroscopy [10, 17]. The mobility obtained from these methods generally reflects the actual (effective) mobility. However, because defect densities can vary significantly across samples, reported mobility values often span a wide range. For example, in the FAPbBr_3_ single crystal, measured mobilities have been reported to range from 29 to 180 cm^2^·V^−1^·s^−1^ [18, 19, 20]. Such variability obscures the intrinsic mobility limits set by fundamental scattering processes, highlighting the need for approaches that can separate intrinsic contributions from defect‐dominated extrinsic effects.

In this work, we quantify the intrinsic carrier mobility determined by acoustic phonon‐carrier (APC) coupling in FA_x_MA_1−x_PbBr_3_ single crystals. Our approach employs time‐domain Brillouin scattering (TDBS) with femtosecond transient reflection (fs‐TR) to measure elastic properties and deformation potentials. First, TDBS probes the generation and propagation of coherent longitudinal acoustic phonons, from which the sound velocities and elastic constants are extracted. Next, the sum of electrons and holes deformation potentials is determined by analyzing the total stresses induced by different excitation photon energies. Subsequently, the difference between the electron and hole deformation potentials was extracted by analyzing the relationship between the bandgap and the unit‑cell volume. Following this, the individual deformation potentials for electrons and holes were determined separately. Using deformation‑potential theory together with our experimentally derived elastic constants and deformation potentials, we calculate the intrinsic electron and hole mobilities governed by APC scattering in FA_x_MA_1−x_PbBr_3_ single crystals. Finally, we analyze and discuss the relationships among elastic properties, deformation potentials, intrinsic mobilities, and FA composition.

## 2 Results and Discussion

**FIGURE 1:**
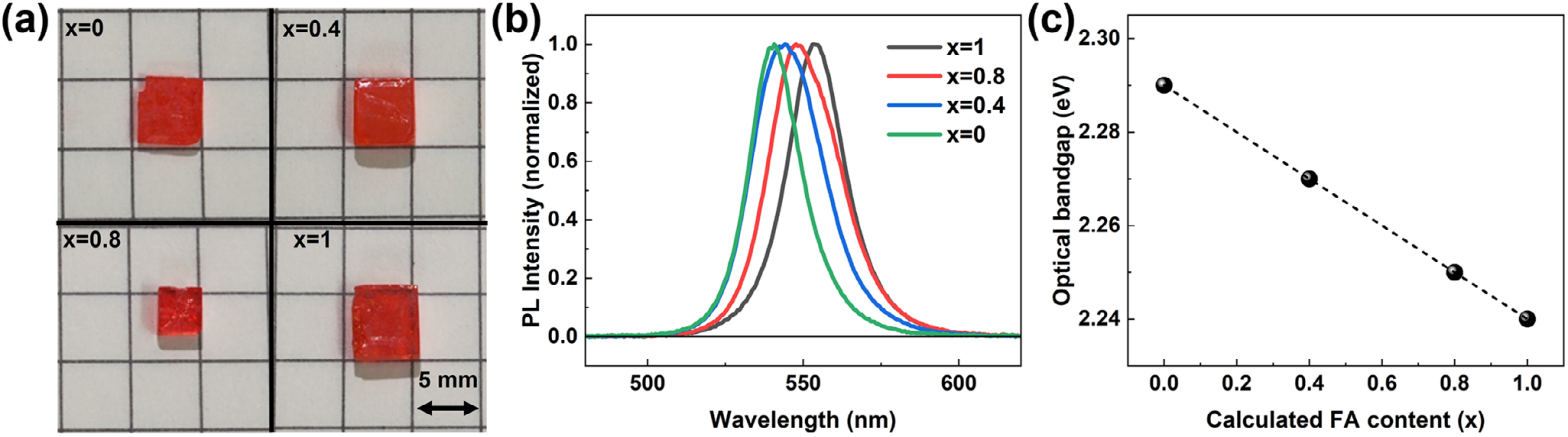
(a) Pictures of FA_x_MA_1−x_PbBr_3_ single crystals. (b) Normalized PL emission for FA_x_MA_1−x_PbBr_3_ single crystals. (c) Correlation between optical bandgap and FA content of FA_x_MA_1−x_PbBr_3_ single crystals.

FA_x_MA_1−x_PbBr_3_ single crystals in different FA contents were fabricated by inverse temperature crystallization, and the crystal pictures are shown in Figure 1a. All samples examined in this work are large bulk single crystals, with lateral dimensions exceeding 2 mm and a thickness of approximately 1 mm. In order to obtain the FA content of FA_x_MA_1−x_PbBr_3_ single crystals, we performed XRD measurements (Figure S1). Through Vegard's law [21], the FA contents were determined as x = 0, 0.4, 0.8, and 1 for all measured FA_x_MA_1−x_PbBr_3_ crystals (see Figure S1 and Table S1). Steady state photoluminescence (PL) measurements were conducted to understand the emission properties of FA_x_MA_1−x_PbBr_3_ single crystals, as shown in Figure 1b. All crystals exhibit a pronounced emission peak, which can be attributed to the band‐gap emissions [22]. As shown in Figure 1c, the peak energy exhibits a near‐linear relationship with FA content (x), corresponding to bandgaps of 2.29 eV (x = 0), 2.27 eV (x = 0.4), 2.25 eV (x = 0.8), and 2.24 eV (x = 1).

To evaluate the quality of our MAPbBr_3_ single crystal, we compared its PL linewidths with values reported for single crystals and polycrystalline thin films in the literature (see Section 2, Supporting Information). The full width at half maximum (FWHM) of the PL peaks for our crystals is narrower than that of a MAPbBr_3_ single crystal with a trap density of 6.2 × 10^9^ cm^−3^ [23]. This indicates that the MAPbBr_3_ crystals in this work possess lower trap densities. To further examine the trap densities and carrier trapping process in FA_x_MA_1−x_PbBr_3_ single crystals, we conducted time‑resolved microwave conductivity (TRMC) measurements, which are sensitive to the change of photoconductivity. We observed that all crystals exhibit long photoconductivity lifetime, and the TRMC decay slows as the FA content (x) increases (Figure S2). This trend suggests that trap densities, which strongly affect photoconductivity and carrier mobility, decrease with higher FA fractions.

In semiconductors, both intrinsic and extrinsic scattering mechanisms reduce carrier mobility. For ideal, nonpolar semiconductor crystalline materials, extrinsic scattering can be neglected. In such systems, lattice acoustic vibrations play a critical role on electron and hole mobility, as explained by the deformation potential (DP) theory [24]. According to DP theory, the carrier mobility governed by APC coupling is given by [24, 25, 26]

(1) $$\mu =\frac{{\left(8\pi \right)}^{1/2}\hbar^{4}eC}{3{\left(m^{*}\right)}^{5/2}{\left(k_{B}T\right)}^{3/2}d^{2}}\;$$

where *C* and *d* are the elastic constant and deformation potential along the relevant crystal axis, ℏ is the reduced Planck constant, *e* is the elementary charge, *k*_B_ the Boltzmann's constant, *T* the temperature, m* the effective mass (electron or hole). As shown in Equation (1), effective mass, elastic constant, and deformation potential are the critical parameters for determining APC coupling‐governed carrier mobility.

**FIGURE 2:**
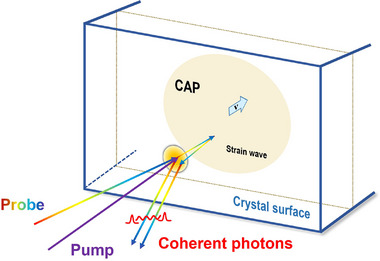
Schematic of strain generation, propagation, and subsequent detection by TR spectroscopy in FA_x_MA_1−x_PbBr_3_ single crystals.

In TDBS experiments, coherent acoustic phonons (CAP) can be generated by exciting perovskite single crystals with a femtosecond laser pulse, and detected by monitoring the transient reflectivity change using time‐delayed probe light [27, 28]. As shown in Figure 2, the CAP wave packet (strain wave) arises from both pump‐induced deformation potential stress and thermal expansion stress. This propagating CAP modifies the local dielectric constant, thereby creating a moving optical interface that reflects the probe light [28, 29, 30]. Since the optical interface travels into the bulk at the acoustic velocity, a time‐linear phase shift develops between light reflected from the surface and from the moving interface. This results in a sinusoidal oscillation of the transient reflectivity at the Brillouin frequency [27, 31]. Analysis of the oscillatory signal enables the determination of the elastic constant and deformation potential of materials.

**FIGURE 3:**
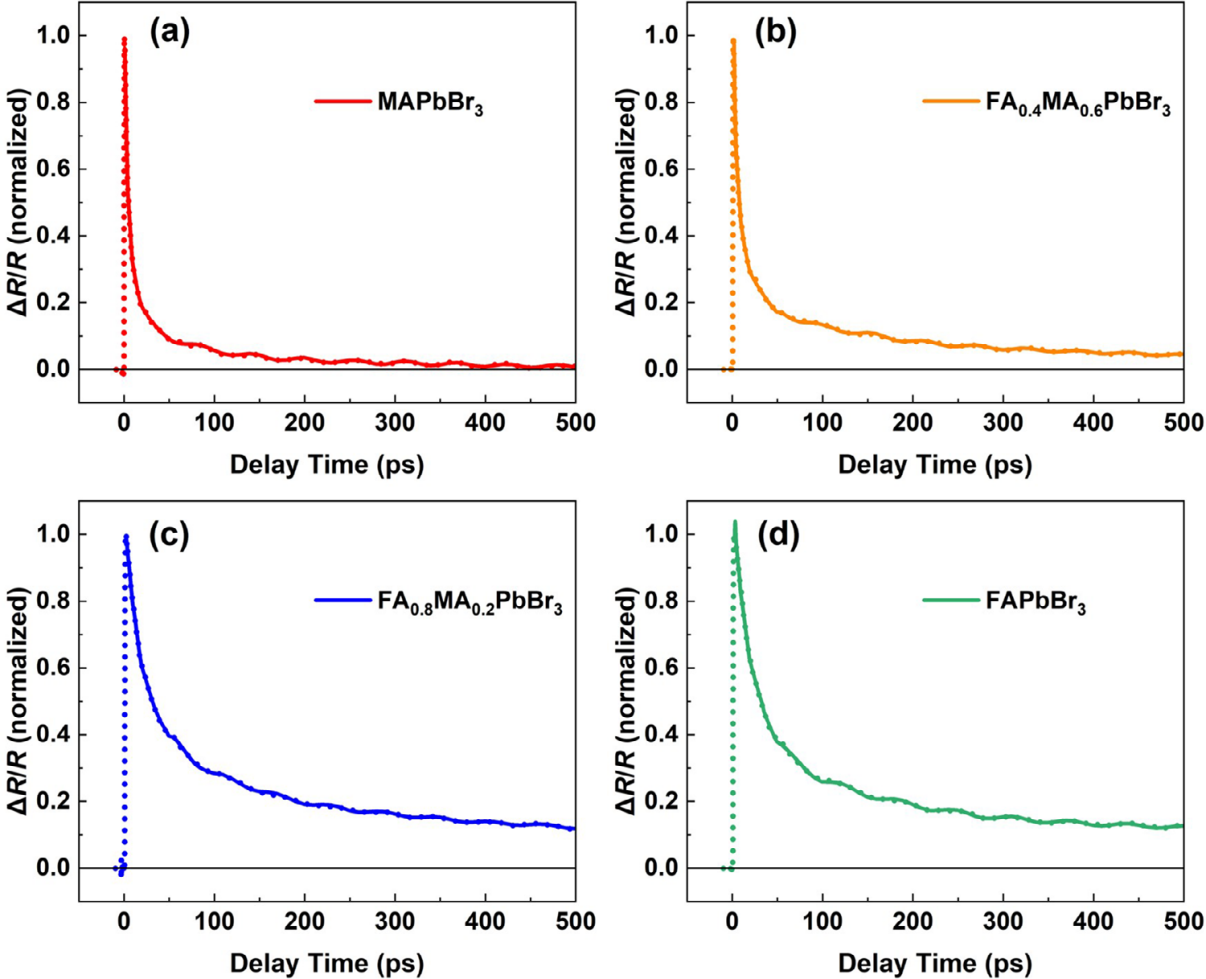
Normalized transient reflection dynamics of (a) x = 0, (b) x = 0.4, (c) x = 0.8, and (d) x = 1 of FA_x_MA_1−x_PbBr_3_ single crystals under 400, 400, 360, and 360 nm excitation, respectively. The solid lines are fitting curve based on the sum of Equation (2) and the background of carrier dynamics. The dot lines represent the experimental data.

**FIGURE 4:**
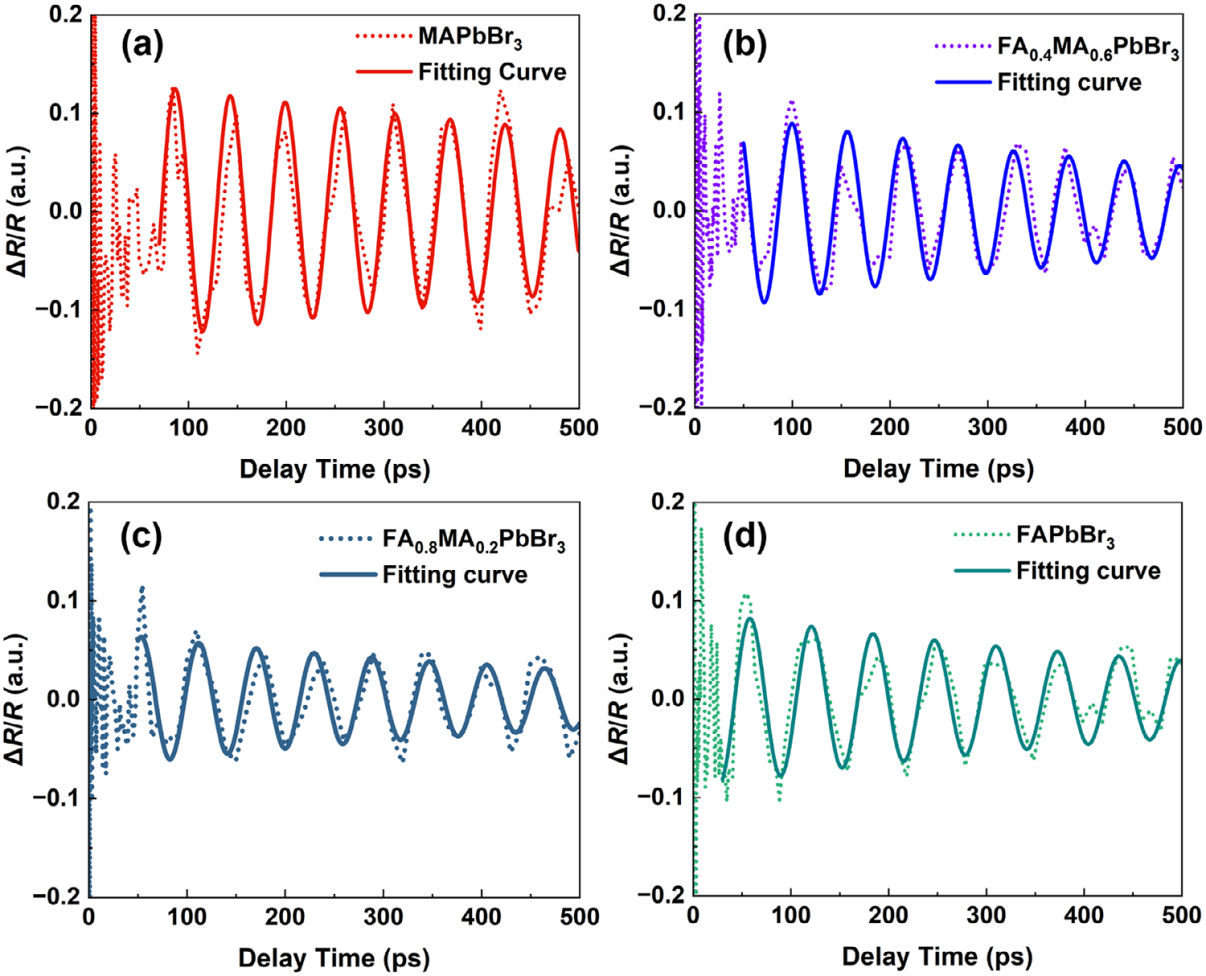
CAP signal of FA_x_MA_1−x_PbBr_3_ single crystals with (a) x = 0, (b) x = 0.4, (c) x = 0.8, and (d) x = 1 at the probe wavelength of 700 nm (x = 0), 717 nm (x = 0.8 and 0.4), and 750 nm (x = 1). Solid lines correspond to fits derived from Equation (2). The excitation fluence for all single crystals is 2.8 × 10^14^ pulse^−1^·cm^−2^.

To determine the elastic constants and the deformation potentials of FA_x_MA_1−x_PbBr_3_ crystals, TDBS experiments based on a TR configuration have been conducted. The excitation and probe laser spot, which defines the effective probed area, is smaller than 1 mm^2^. This is considerably less than the exposed crystal facet area, which is greater than 4 mm^2^. Although the absolute dimensions of individual crystals vary to some extent, all crystals remain substantially larger than the optical probe area. Therefore, the effective measurement region is consistent across all samples, and within this region, the results can be considered independent of sample geometry. Figure 3 displays the TR kinetics of FA_x_MA_1−x_PbBr_3_ (x = 0, 0.4, 0.8, and 1) crystals at room temperature. All the transient reflection signals Δ*R*/*R* in the samples exhibit a sharp rise and a subsequent decay dynamics resulted from carrier recombination and diffusion following photoexcitation [32]. Meanwhile, we observe a CAP‐induced oscillatory signal superimposed on the decaying background, which originates from the change in the phase difference of the coherent photons [27]. By removing the background of carrier dynamics, the CAP signal can be extracted, as shown in Figure 4. The CAP signals of FA_x_MA_1−x_PbBr_3_ single crystals with varied FA contents at the (100) direction all exhibit a gradual decay over time, which could be induced by impurity‐induced phonon scattering [12].

**FIGURE 5:**
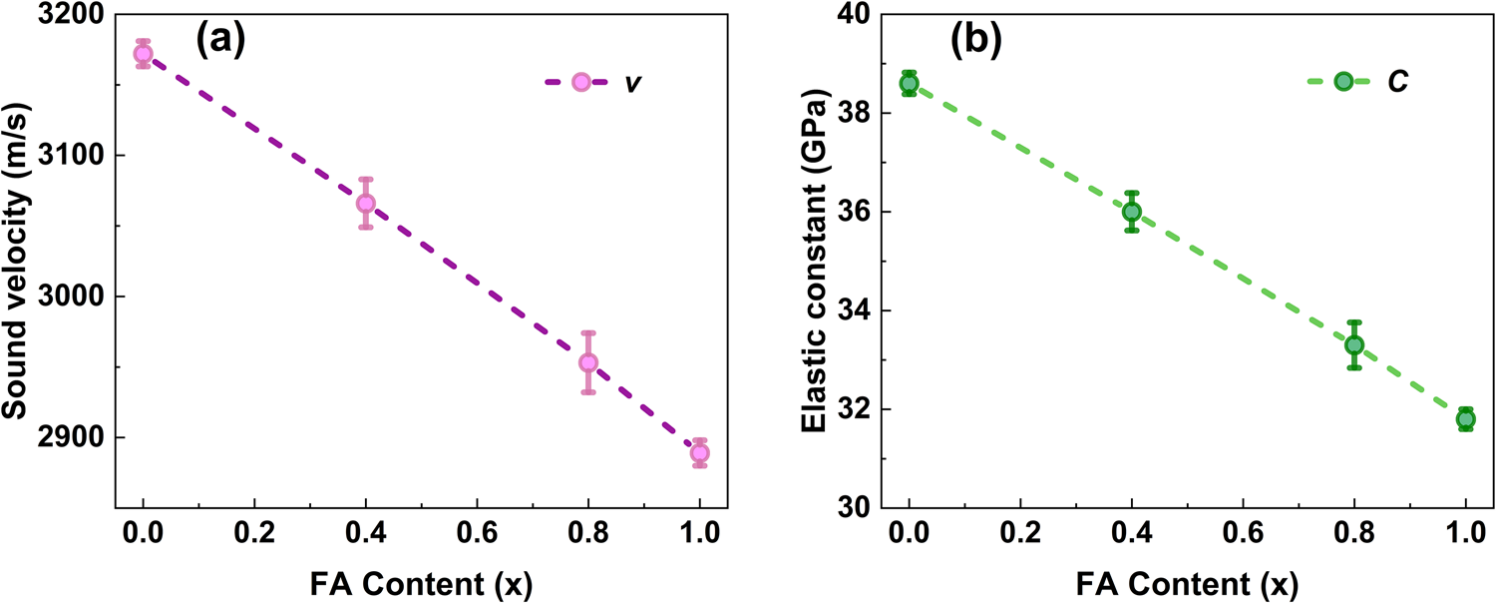
(a) Sound velocity and (b) elastic constant versus FA content in FA_x_MA_1−x_PbBr_3_ single crystals.

To extract the parameters of oscillation signal, the CAP signals near the target probing wavelength were globally fitted with the function below

(2) $$y=A_{B}\;e^{-\frac{t-t_{c}}{\tau_{B}}}\cos\left(\frac{2\pi \left(t-t_{c}\right)}{\tau }\right)$$

where *A_B_* represents the amplitude, *τ*_*B*_ the lifetime, *t_c_* the initial time, and *τ* the period. Complete fitting values are provided in Table S3. From the Brillouin oscillation frequency *f*, the sound velocity *v* can be obtained via [31, 33, 34]

(3) $$v=\frac{f\lambda }{2n}=\frac{\lambda }{2n\tau }\;$$

where *λ* is the probe light wavelength and *n* is the refractive index of the crystal at the probe light wavelength, which is determined by ellipsometry (as shown in Figure S3). Using Equation (3), the sound velocities of 3173 ± 9, 3066 ± 16, 2953 ± 21, and 2889 ± 9 m/s were determined for FA_x_MA_1−x_PbBr_3_ single crystals with FA contents of x = 0, 0.4, 0.8, and 1, respectively. Apparently, the sound velocity along [100] of the crystals decreases with the increase of FA content, as shown in Figure 5a. The elastic constant along the direction of phonon propagation (*C*) is then calculated from the sound velocity using the relation [12]

(4) $$C=v^{2}\rho$$

where *ρ* denotes the mass density. Assuming that the mass density varies linearly with FA content [35], the  *C* value for the corresponding compositions are found to be 38.6 ± 0.2, 36.0 ± 0.4, 33.3 ± 0.5, and 31.8 ± 0.2 GPa (Table S3). Due to low trap densities of crystals in this work, we would not expect much influence of trap on *C*. The elastic constant characterizes the strength of interatomic bonding from the perspective of interatomic forces [12]. A higher elastic constant signifies stronger interatomic bonds. As shown in Figure 5b, the decreasing elastic constant with increasing FA content in FA_x_MA_1−x_PbBr_3_ single crystals indicates a reduction in interatomic bonding strength. This observation aligns with reports of MAPbBr_3_ exhibits stronger hydrogen bonding between MA^+^ cations and halide ions than FAPbBr_3_ does between FA^+^ and halides [36, 37], consistenting with the higher elastic constant we measured for MAPbBr_3_.

To extract the APC governed charge carrier mobility, the deformation potentials of electrons and hole should be determined. As noted earlier, the generation of CAP arises from two main contributions: pump‐induced thermal expansion stress and deformation potential stress. The thermal expansion stress *σ*_TE_ results from the excess energy of photogenerated carriers that is transferred to the lattice, heating it and inducing stress through thermal expansion. It can be expressed as [12, 30, 38].

(5) $$\sigma_{\mathrm{TE}}=-3B\beta N\frac{h\nu -E_{g}}{C_{p}}$$

where *B* denotes the bulk modulus, *β* the coefficient of linear expansion, *N* the density of photoexcited carriers, *h* *ν* the pump light photon energy, *E*_g_ the band gap, and *C*_p_ the heat capacity. In contrast, the deformation potential stress σ_DP_ mainly originates from the variation of atom binding energies induced by photoexcitation. The bonding between atoms changes when electrons are excited from the valence band to the conduction band, resulting in the generation of the deformation potential stress *σ*_DP_. The stress *σ*_DP_ can be described as [12, 30, 38].

(6) $$\sigma_{\mathrm{DP}}=-N\left(d_{e}+d_{h}\right)$$

where *d*_e_ and *d*_h_ denote the deformation potentials of electrons and holes, respectively. As the deformation potential stress is proportional to the carrier concentration and the sum of the deformation potentials of electrons and holes, the contribution of *σ*_DP_ to CAP should not change at a constant photogenerated carrier concentration, whereas *σ*_TE_ varies with the pump photon energy. The amplitudes *A* of Brillouin scattering signal is proportional to the sum of *σ*_TE_ and *σ*_DP_, that is

(7) $$A\propto \sigma_{\mathrm{TE}}+\sigma_{\mathrm{DP}}=-3B\beta N\frac{h\nu -E_{g}}{C_{p}}-N\left(d_{e}+d_{h}\right)$$

By varying the photon energy of pump, the relative contributions of thermoelastic and deformational stresses to the CAP can be tuned, allowing the sum of deformation potential (*d*_e_+*d*_h_) for FA_x_MA_1−x_PbBr_3_ single crystals to be extracted (Sections 6–8, Supporting Information). It should be noted that the influence of penetration depths at different excitation wavelengths on Brillouin scattering amplitude at the detected phonon frequency should be considered and corrected when extracting the sum of deformation potential (Sections 5–6, Supporting Information).

**FIGURE 6:**
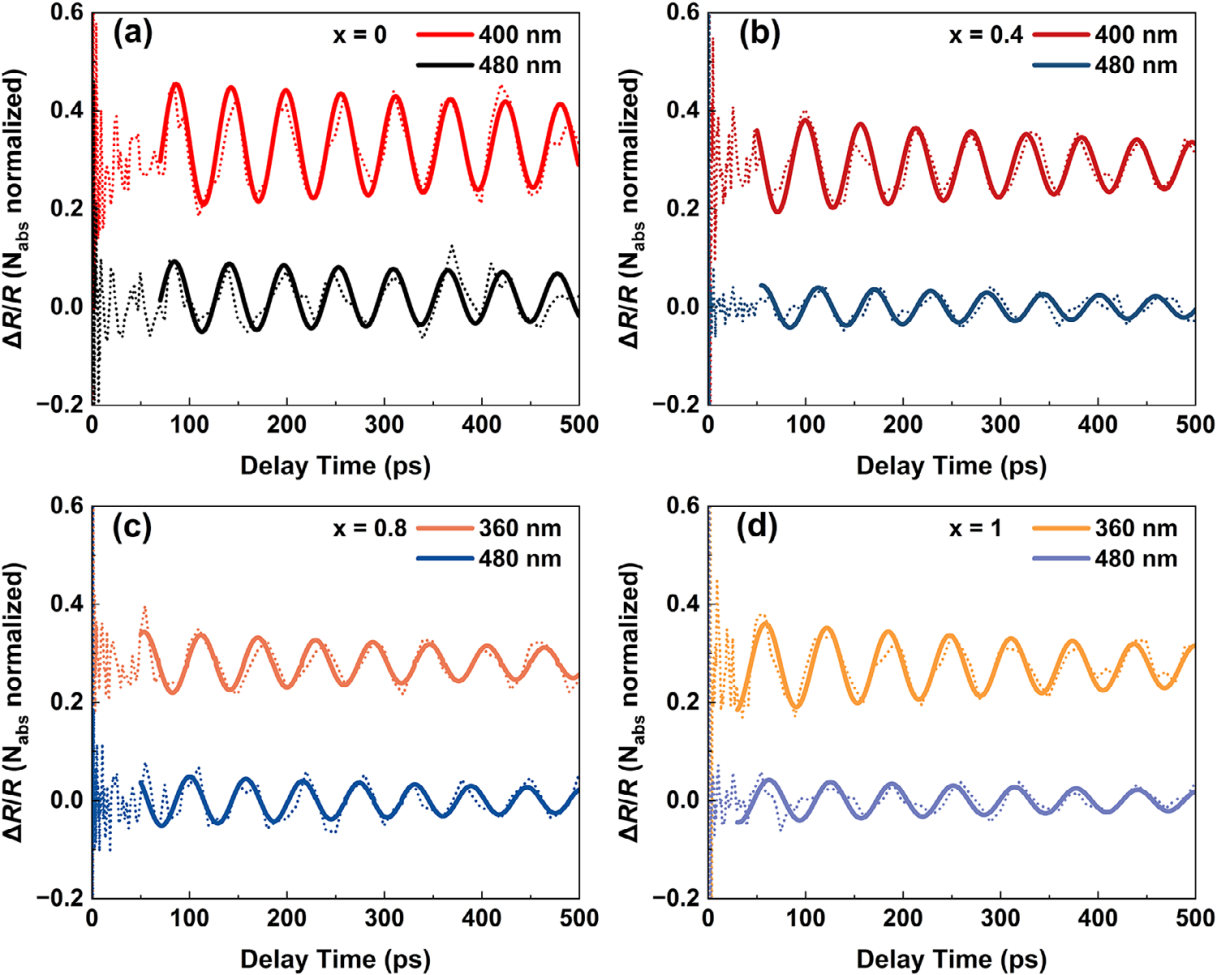
CAP signal of FA_x_MA_1−x_PbBr_3_ single crystals with (a) x = 0, (b) x = 0.4, (c) x = 0.8, and (d) x = 1 at a probe wavelength of 700 nm (x = 0), 717 nm (x = 0.8 and 0.4), and 750 nm (x = 1) under the indicated excitation wavelength. Solid lines correspond to fits derived from Equation (2), and detailed fitting values are listed in Table S5. The signals have been corrected by considering the absorption at different excitation photon energies.

Figure 6 shows Brillouin scattering signals of the FA_x_MA_1−x_PbBr_3_ single crystals under different excitation wavelengths. These signals were corrected by absorption corresponding to the excitation photon energies listed in Table S4. Detailed fitting parameters are provided in Table S5. We observe that the Brillouin scattering amplitudes of all crystals increase with the decreasing excitation wavelength, as higher excitation photon energy (shorter wavelength) provides more excess energy and thus strengthens thermal expansion stress. Based on the Brillouin scattering amplitudes at different excitation photon energies, and substituting the bandgap and literature‐reported bulk modulus, linear expansion coefficient, and heat capacity into Equation (7) [39, 40], the sum of deformation potentials (*d*_e_+*d*_h_) can be determined (Section 8, Supporting Information).

According to original deformation potential theory, which links energy level shifts to volumetric strain, the potentials for electrons (*d*_e_) and holes (*d*_h_) are defined as [41].

(8) $$d_{e}=V\frac{dE_{\mathrm{CBM}}}{dV},\;d_{h}=V\frac{dE_{\mathrm{VBM}}}{dV}$$

where *E*_CBM_ and *E*_VBM_ are the energies at the conduction band minimum and valence band maximum, respectively, and *V* is the unit cell volume.

Consequently, their difference relates directly to the bandgap (*E*_g_ = *E*_CBM_  − *E*_VBM_):

(9) $$d_{e}-d_{h}=V\;\frac{d\left(E_{\mathrm{CBM}}-E_{\mathrm{VBM}}\right)}{\mathrm{dV}}=V\frac{dE_{g}}{\mathrm{dV}}$$

This leads to

(10) $$dE_{g}=\left(d_{e}-d_{h}\right)\frac{1}{V}\mathrm{dV}$$

and upon integration by assuming (*d*_e_−*d*_h_) is a constant

(11) $$E_{g}=\left(d_{e}-d_{h}\right)\ln V+b$$

**FIGURE 7:**
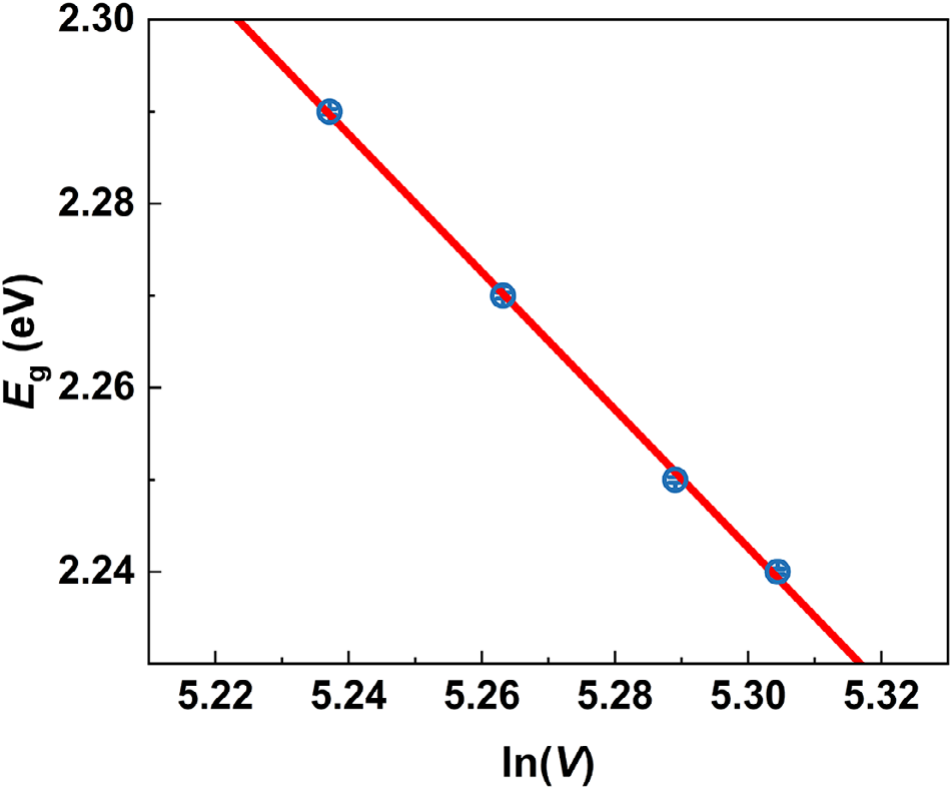
The correlation between bandgap (*E*_g_) and ln(*V*). The solid line is the linear fitting curve.

**FIGURE 8:**
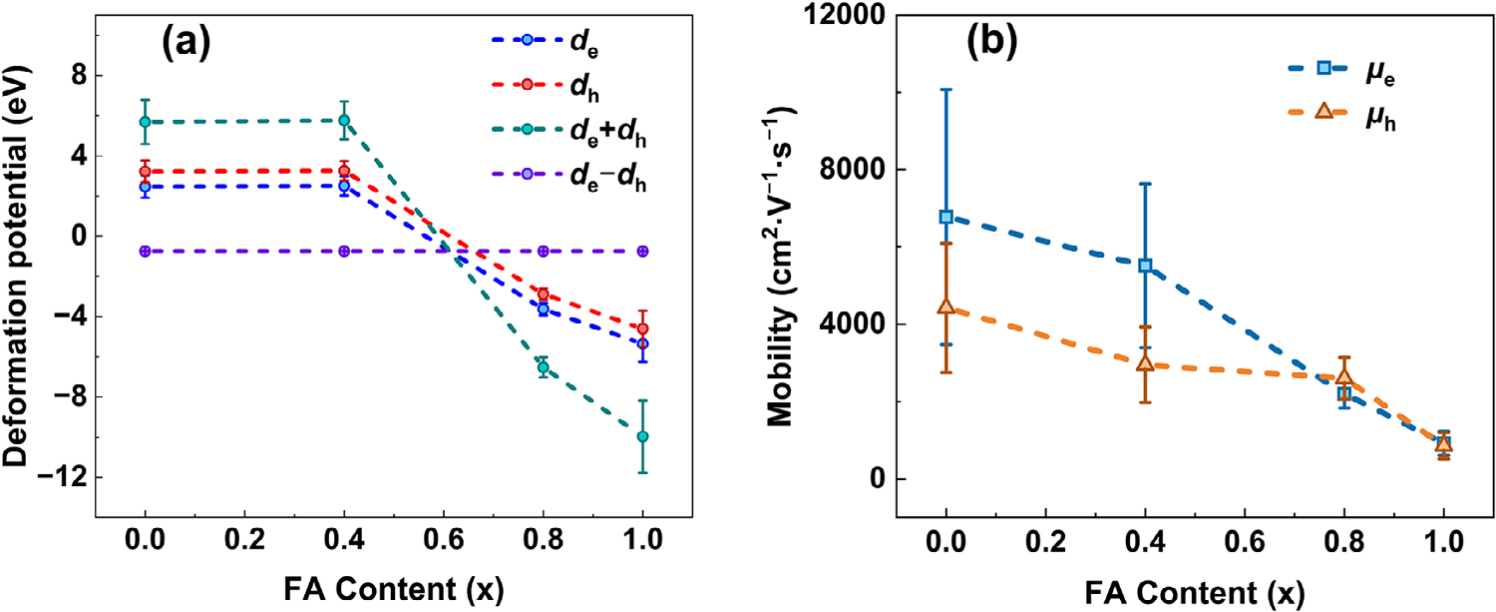
Variation of (a) deformation potential and (b) APC‐governed intrinsic carrier mobility with FA composition (x) in FA_x_MA_1−x_PbBr_3_ single crystals.

Therefore, by knowing the correlation between bandgap *E*_g_ and the unit cell volume *V*, the slope (*d*_e_ *−d*_h_) can be thus obtained by linear fitting, as shown in Figure 7 and Table S7. The observed linear dependence suggests unit cell volume *V* is the primary factor determining the change of *E*_g_with variation of FA composition. By combining this slope (*d*_e_−*d*_h_) with the sum deformation potentials (*d*_e_+*d*_h_) obtained from Brillouin scattering measurements, we can separately extract the individual deformation potentials *d*_e_ and *d*_h_ (Figure 8a; Table S7). Using these values, APC governed intrinsic electron and hole mobilities are then determined (Figure 8b).

It should be noted that the raised method for determining APC coupling governed intrinsic carrier mobility requires precise evaluation of both the elastic constant corresponding to the longitudinal acoustic phonons, and the deformation potentials of electron and hole. All perovskite crystals studied here are in the cubic phase at room temperature. In our TDBS experiments, with probing along the [001] crystallographic direction, the measured elastic constant corresponds directly to that of the longitudinal acoustic phonon mode. However, for highly anisotropic crystal structures, such as the tetragonal phase, the frequencies obtained from TDBS measurements along different crystallographic directions may include not only the longitudinal mode but also transverse modes. For example, in tetragonal MAPbI_3_ at room temperature, TDBS measurements from the (112) plane can reveal frequencies, and the corresponding sound velocities and elastic constants, associated with shear, quasi shear, and quasi longitudinal phonons [42]. Therefore, when applying deformation potential theory, it is essential to carefully identify the frequency corresponding to the longitudinal mode and the elastic constant derived from it. Besides, in some mixed anion (or mixed cation) perovskite crystals, alloy scattering can broaden the phonon peaks in the TDBS signal, increasing the difficulty of accurately extracting sound velocities and elastic constants.

Furthermore, our methodology involves extracting the sum of deformation potentials of electrons and holes (*d*_e_+*d*_h_). This approach relies on the assumption that defect‐related trapping occurs on a timescale much longer than the establishment of the initial Brillouin scattering amplitude, thus minimizing any influence of energy released during carrier trapping on the measured signal. Consequently, it is essential for the studied crystals to exhibit low trap densities. In our case, TRMC measurements suggests charge trapping time of the crystals is far longer than the time required for the establishment of the initial Brillouin scattering amplitude (<100 ps). Hence, the carrier trapping process of crystals has little influence on the initial amplitude. In cases where the crystal possesses a high defect‐state density, a quantitative assessment of the trap types and the associated energy release during carrier trapping would be required for the extraction of *d*_e_ and *d*_h_ [12]. Additionally, for extracting (*d*_e_ *−d*_h_) via Equation (11), the series of crystals employed should share the same type of crystal structure, and the lattice parameter is the key factor in determining the change of bandgap.

The deformation potential quantifies the energy shift of conduction and/or valence band edges under applied strain, exhibiting proportionality to the induced stress [24]. A larger absolute deformation potential indicates a greater stress generation under identical carrier concentration, reflecting a higher sensitivity of the electronic band structure to strain and consequently lower lattice stability [43]. As presented in Figure 8a, the absolute value of deformation potentials of both electrons and holes generally increase with FA content (x). This trend may be attributed to larger ionic radius of FA^+^ and its reduced hydrogen‐bonding capacity compared to MA^+^, which collectively enhance lattice distortion, leading to greater band edge fluctuations under strain. Using the obtained deformation potential, elastic constant, and effective mass into Equation 1, we estimated the electron and hole mobilities in perovskite governed by acoustic phonon scattering (Section 9, Supporting Information). As shown in Figure 8b, both APC‐governed intrinsic electron and hole mobilites decreases with increasing FA content. While MAPbBr_3_ exhibits both intrinsic electron and hole mobilities exceeding 4000 cm^2^·V^−1^·s^−1^, FAPbBr_3_ shows mobilities below 1000 cm^2^·V^−1^·s^−1^. This demonstrates that the detrimental impact of acoustic phonon scattering on carrier mobility becomes progressively stronger at higher FA contents.

Notably, for both electrons and holes, the extracted APC coupling governed mobilities in many FA_x_MA_1−x_PbBr_3_ single crystals are on the order of thousands of cm^2^·V^−1^·s^−1^. These values substantially exceed carrier mobilities typically measured by time‐of‐flight (TOF), space‐charge‐limited current (SCLC), and time‐resolved THz spectroscopy (TRTS) in perovskites, which are often below 200 cm^2^·V^−1^·s^−1^ [18, 44, 45]. As an intrinsic factor determining the upper mobility limit, the APC coupling governed mobility represents the theoretical maximum achievable when this scattering mechanism dominates. However, in perovskite materials, where multiple scattering mechanisms coexist (such as carrier‐phonon and carrier‐defect scattering), the reported mobility reflects the combined effect, often dominated by the scattering other than acoustic phonon‐carrier scattering [18, 44, 45]. In polycrystalline perovskite thin films and in single crystals with high defect densities, carrier‐defect scattering often dominates the actual (or effective) carrier mobility. For example, the reported mobility of FAPbBr_3_ polycrystalline films can span a wide range from 6.9 × 10^−4^ to 21.4 cm^2^·V^−1^·s^−1^ [45, 46, 47], depending on the density of defect states. Even for FAPbBr_3_ single crystals, the carrier mobility can vary, for instance, between 29 and 203 cm^2^·V^−1^·s^−1^ [18, 19, 20, 48].

Beyond carrier‐defect scattering, longitudinal optical (LO) phonon scattering has been identified as the dominant mechanism governing carrier mobility at room temperature in lead halide perovskites [49, 50]. Both polaron‐based and LO‑phonon‑limited charge‑transport models highlight the critical role of the Fröhlich interaction between carriers and LO phonons. Based on these models, carrier mobility can be evaluated using the Feynman‐Ōsaka general equations or by analyzing the temperature dependence of photoluminescence linewidth broadening [49, 50]. The resulting mobilities typically fall in the range of ∼100–300 cm^2^·V^−1^·s^−1^, reflecting the intrinsic limit imposed by Fröhlich scattering. In contrast, our TDBS experiments, combined with deformation potential theory, are designed to quantify the scattering due to acoustic phonons. This could be a weaker scattering mechanism compared to LO‐phonon coupling. The high mobility we extract would represent the intrinsic upper limit that would be attainable if scattering from LO phonons (and other extrinsic factors) could be completely suppressed.

## 3 Conclusion

In this work, we quantify the APC coupling determined intrinsic carrier mobilities of FA_x_MA_1−x_PbBr_3_ single crystals by measuring elastic properties and deformation potentials using time‐domain Brillouin scattering (TDBS) based on femtosecond transient reflection (fs‐TR) techniques. Leveraging the relationship between oscillatory signals and the material's elastic constant and sound velocity, we experimentally determined these parameters. Furthermore, by analyzing the relationship between the total stresses induced by the pump laser at different photon energies, we successfully extracted the total deformation potentials for FA_x_MA_1−x_PbBr_3_ single crystals. It was found that both the sound velocity and elastic constant in FA_x_MA_1−x_PbBr_3_ single crystals decrease with the increasing FA content. Applying deformation potential (DP) theory to our experimentally determined elastic constants and deformation potentials, we determined the intrinsic carrier mobilities for FA_x_MA_1−x_PbBr_3_ single crystals. These results demonstrate that intrinsic carrier mobilities of both electrons and holes, dominated by the carrier‐acoustic phonon coupling, decrease with increasing FA content (x) in FA_x_MA_1__−__x_PbBr_3_ single crystals. This study deepens the understanding of intrinsic carrier transport characteristics and provides fundamental insights for the rational design of high‐performance perovskite materials.

## 4 Experimental Section/Methods

### 4.1 Chemicals and Reagents

N, N‐dimethylformamide (DMF, 99.5%), formamidinium bromide (FABr, 99%), lead bromide (PbBr_2_, 98%), and methylammonium bromide (MABr, 98%) were purchased from Aladdin. All chemicals were utilized as received without further purification.

### 4.2 Growth of FA_x_MA_1−x_PbBr_3_ Perovskite Single Crystals (SCs)

FA_x_MA_1−x_PbBr_3_ SCs were fabricated according to the parameters detailed in Table S8. Briefly, solutes were dissolved in the specified solvent at the molar ratios provided in Table S8. This mixture was stirred (500 rpm, 24 h, room temperature) until complete dissolution was achieved. The resulting solution was then filtered through a nylon filter (0.22 µm pore size) to obtain a clear precursor solution. Seed crystals (1∼2 mm) were subsequently grown by heating this precursor solution at 60°C for 72 h. Finally, the precursor solution was filtered, and a selected seed crystal was placed in. Growth then proceeded at 60°C until the crystal attained a size of 2∼5 mm. The detailed parameters of FA_x_MA_1−x_PbBr_3_ SCs were shown in Table S8.

### 4.3 Time Domain Brillouin Scattering (TDBS) Measurements

The measurements were measured by the HARPIA‐TA system (Light Conversion). The excitation source was a femtosecond laser (Pharos, Light Conversion), emitting 190 fs pulses at 1030 nm with a 100 kHz repetition rate. The fundamental 1030 nm beam was then split into two separate paths. The first beam is sent to an optical parametric amplifier (Orpheus‐HP, Light Conversion), whose output serves as the HARPIA‐TA system's pump source. The second beam generates super‐continuum white light, functioning as the probe light used in differential reflection measurements. Both of the pump and probe beams are focused on the same place of the sample. The time delay is modulated with a mechanical delay line in the probe path. In TDBS experiments, coherent longitudinal acoustic phonons (CAP) were generated by exciting perovskite single crystals with femtosecond laser pulse, and were detected by monitoring the transient reflectivity change with time‐delayed probe light.

## Funding

This work was supported by the National Natural Science Foundation of China (22473033), Guangdong Basic and Applied Basic Research Foundation (2019A1515010783), Basic and Applied Basic Research Program of Guangzhou (202102010443), and Guangzhou University (ZH2023005).

## Conflicts of Interest

The authors declare no conflict of interest.

## Supporting information




**Supporting File**: advs74327‐sup‐0001‐SuppMat.docx

## Data Availability

The data that support the findings of this study are available from the corresponding author upon reasonable request.
