# Supplementary material for: Charge‐Acoustic Phonon Coupling Determined Carrier Transport Properties in FAxMA1−xPbBr3 Perovskite Single Crystals
Source: Adv Sci (Weinh). 2026 Feb 13;13(22):e24220. doi: 10.1002/advs.202524220 (PMC13088333; doi:10.1002/advs.202524220)
Supplement: Supplementary file 1 — Supporting File: advs74327‐sup‐0001‐SuppMat.docx [file ADVS-13-e24220-s001.docx]

Supporting Information

Charge-acoustic phonon coupling determined carrier transport properties in FA_x_MA_1−x_PbBr_3_ perovskite single crystals

Wei Zhang^1,*^, Zheng Zou^1^, Tiehui Wu^1^, Zijie Xiao^2,*^, Dan Feng^1,3^, Jianbin Zhong^1^, Xianshao Zou^4^, and Ningjiu Zhao^3,*^

W. Zhang, Z. Zou, T. Wu, D. Feng, J. Zhong
School of Physics and Materials Science, Guangzhou University, Guangzhou 510006, China E-mail: wzhang@gzhu.edu.cn

Z. Xiao
Max Planck Institute for Polymer Research, Mainz 55128, Germany

E-mail: xiaoz@mpip-mainz.mpg.de

X. Zou
Qingdao Innovation and Development Center, Harbin Engineering University, Qingdao 266000, China

D. Feng, N. Zhao
Songshan Lake Materials Laboratory, Dongguan, Guangdong 523808, China

E-mail: zhaonj@dicp.ac.cn

**Keywords: carrier mobility, charge transport, elastic modulus, perovskite single crystal**

**Content**

[**Section 1 FA content and optical bandgap of FA_x_MA_1−x_PbBr_3_ single crystals 3**](#_Toc221362764)

[**Section 2 PL linewidths and trap densities in this work and literatures 4**](#_Toc221362765)

[**Section 3 TRMC kinetics of FA_x_MA_1−x_PbBr_3_ crystals 5**](#_Toc221362766)

[**Section 4 The calculation of elastic constants of FA_x_MA_1−x_PbBr_3_ single crystals 6**](#_Toc221362767)

[**Section 5 The refractive index *n* and the extinction coefficient *k* of FA_x_MA_1−x_PbBr_3_ single crystals 7**](#_Toc221362768)

[**Section 6 Extraction of deformation potentials 8**](#_Toc221362769)

[**Section 7 Fitting parameters of Brillouin scattering signal under different excitation wavelengths for FA_x_MA_1−x_PbBr_3_ single crystals 11**](#_Toc221362770)

[**Section 8 Parameters related to FA_x_MA_1−x_PbBr_3_ single crystals deformation potential extraction 12**](#_Toc221362771)

[**Section 9 Parameters for intrinsic carrier mobility calculation in FA_x_MA_1−x_PbBr_3_ single crystals 13**](#_Toc221362772)

[**Section 10 Growth conditions of FA_x_MA_1−x_PbBr_3_ single crystals with varied FA content 14**](#_Toc221362773)

[**REFERENCES 15**](#_Toc221362774)

# Section 1 FA content and optical bandgap of FA_x_MA_1−x_PbBr_3_ single crystals

FA_x_MA_1−x_PbBr_3_ single crystals with different FA contents were grown by the inverse temperature crystallization method. To determine the FA content of FA_x_MA_1−x_PbBr_3_ single crystals, we performed XRD measurements on FA_x_MA_1−x_PbBr single crystals, as shown in Figure S1. The lattice parameters of the crystal can be calculated by the Bragg diffraction equation ($2dsin \theta=n\lambda$) with the 2$\theta$ position at (200), as shown Table S1. According to Vegard’s law, the lattice parameter (*a*) varies quasi-linearly with the FA composition (x).^[1]^ The FA content can be determined from the quasi-linear relationship with lattice parameters:

$FA Content \left( x \right)=\frac{a_{{FA}_{x}{{MA}_{1-x}PbBr}_{3}}-a_{{MAPbBr}_{3}}}{a_{{FAPbBr}_{3}}-a_{{MAPbBr}_{3}}}$ (S1)

where $FA Content \left( x \right)$ is the molar fraction of FA in FA_x_MA_1−x_PbBr_3_; $a_{{FA}_{x}{{MA}_{1-x}PbBr}_{3}}$ is the lattice parameter of the crystal with FA composition of *x*; $a_{{MAPbBr}_{3}}$ is the lattice parameter of MAPbBr_3_; and $a_{{FAPbBr}_{3}}$ is the lattice parameter of FAPbBr_3_. The values are shown in Table S1.


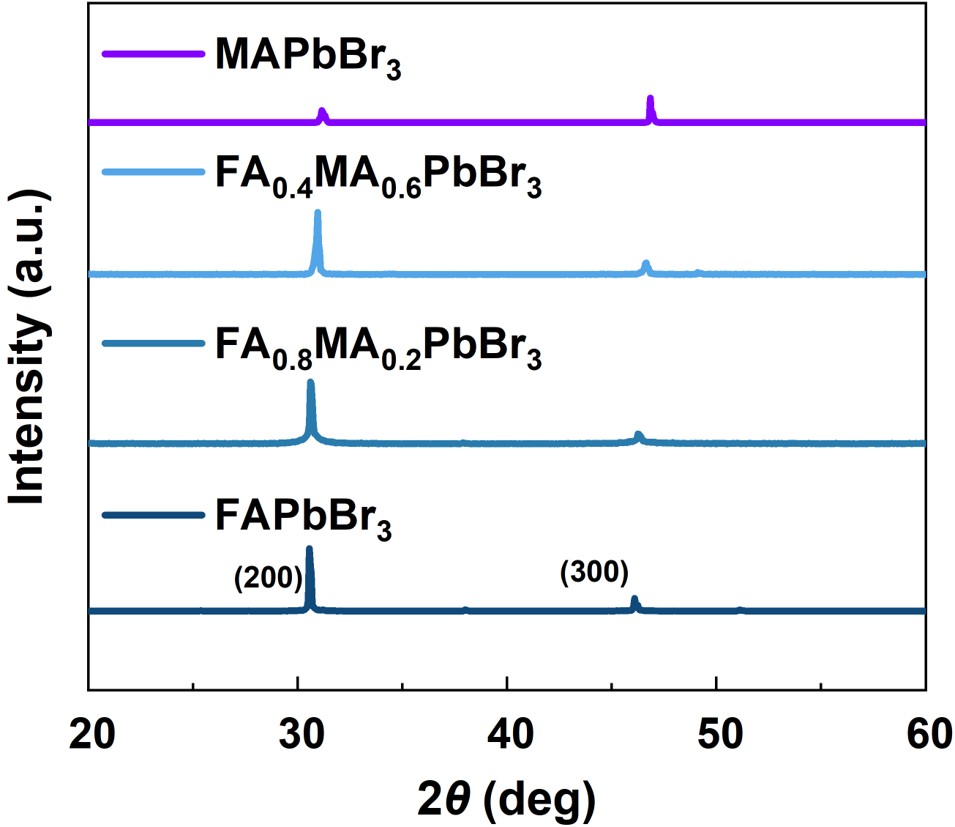


Figure S1. XRD of FA_x_MA_1−x_PbBr_3_ single crystals in the range of 20°<2*θ*<60°.

Table S1. Lattice parameters of FA_x_MA_1−x_PbBr_3_ single crystals with varied FA content.

| FA Content [X] | Phase | *a* [Å] |
| --- | --- | --- |
| 0 | Cubic | 5.73 |
| 0.4 | Cubic | 5.78 |
| 0.8 | Cubic | 5.83 |
| 1 | Cubic | 5.86 |

# Section 2 PL linewidths and trap densities in this work and literatures

Table S2 PL full width at half-maximum (FWHM) and trap density of different perovskite single crystal and thin film.

| Sample | PL FWHM (nm) | Trap density (cm^−3^) | Ref. |
| --- | --- | --- | --- |
| MAPbBr_3_ single crystals | ~ 19.6 | / | This work |
|  | 21.3 | 6.2 × 10^9^ | [2] |
| MAPbBr_3_ thin films | ~ 35.7 | / | [3] |
|  | 30.6 | / | [4] |
|  | / | ~ 10^16^ | [5] |
|  | / | ~ 10^16^ | [6] |
|  | / | 10^17^ | [7] |
| FAPbBr_3_ single crystals | ~ 21.7 | / | This work |
|  | 19.5 | / | [8] |
|  | 24.0 | / | [9] |
|  | / | 9.6 × 10^9^ | [10] |
|  | / | 4.1 × 10^9^ | [11] |
| FAPbBr_3_ thin films | 26.6 | / | [12] |
|  | 25.0 | / | [13] |
|  | / | ~ 10^15^-10^16^ | [14] |
|  | / | 2.8 × 10^16^ | [15] |
| FAPbBr_3_ single crystal thin films | / | 3.76 × 10^8^ | [16] |
| FA_0.4_MA_0.6_PbBr_3_ single crystals | ~ 26.7 | / | This work |
| FA_0.8_MA_0.2_PbBr_3_ single crystals | ~ 26.5 | / | This work |

# Section 3 TRMC kinetics of FA_x_MA_1−x_PbBr_3_ crystals


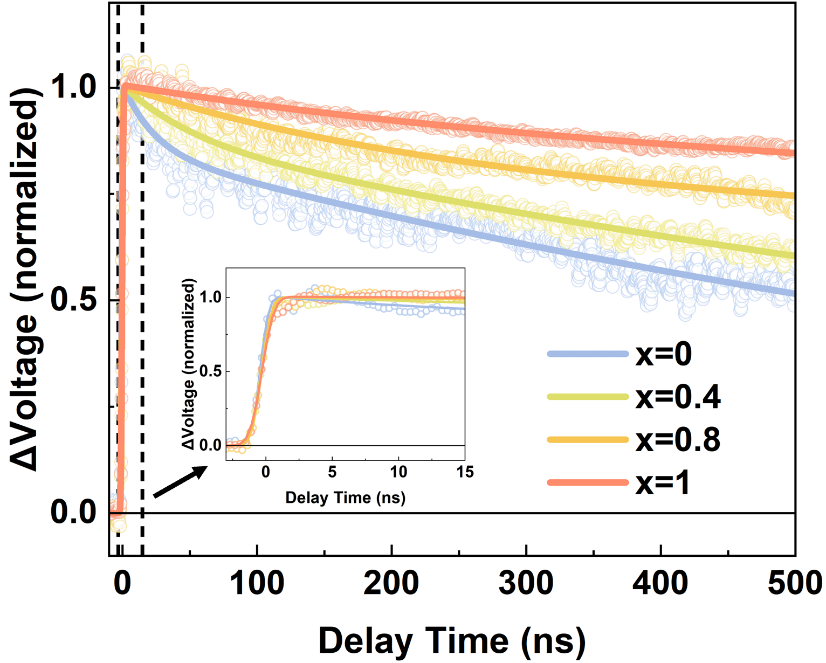


Figure S2. TRMC kinetics of FA_x_MA_1−x_PbBr_3_ crystals. The measurement were carried out at a fluence of 3.15×10^12^ pulse^−1^·cm^−2^ after 400 nm photoexcitation.

# Section 4 The calculation of elastic constants of FA_x_MA_1−x_PbBr_3_ single crystals

Table S3. Parameters related to the extraction of the elastic constants of FA_x_MA_1−x_PbBr_3_ single crystals.

| x | Probe  [nm] | $t_{c}$  [ps] | $\tau$[ps] | $\tau_{B}$  [ps] | $A_{B}$  [×10^−2^] | $f$  [GHz] | $n$ | $v$  [m/s] | $\rho$  [kg/m^3^] | $C$  [GPa] |
| --- | --- | --- | --- | --- | --- | --- | --- | --- | --- | --- |
| 0 | 700 | 30 | 56.0($\pm$0.2) | 990 | 13.0 ($\pm$0.3) | 17.9 | 1.97 | 3173($\pm$9) | 3834^[17]^ | 38.6($\pm$0.2) |
| 0.4 | 717 | 37 | 56.2($\pm$0.3) | 599 | 9.7($\pm$0.2) | 17.8 | 2.08 | 3066($\pm$16) | 3830 | 36.0($\pm$0.4) |
| 0.8 | 717 | 45 | 58.0($\pm$0.4) | 595 | 6.3 ($\pm$0.3) | 17.2 | 2.09 | 2953($\pm$21) | 3817 | 33.3($\pm$0.5) |
| 1 | 750 | 55 | 62.1($\pm$0.2) | 596 | 8.2($\pm$0.3) | 16.1 | 2.09 | 2889($\pm$9) | 3807^[17]^ | 31.8($\pm$0.2) |

The elastic constants of FA_x_MA_1−x_PbBr_3_ single crystals were calculated via $C=\nu^{2}\rho$ using literature values for the mass density *ρ*. For compositions where *ρ* was not reported in literature (x = 0.4 and 0.8), a linear dependence of *ρ* on FA content was assumed.

# Section 5 The refractive index *n* and the extinction coefficient *k* of FA_x_MA_1−x_PbBr_3_ single crystals





Figure S3. The refractive index $n$ and the extinction coefficient $k$ for (a) x=0, (b) x= 0.4, (c) x=0.8, and (d) x=1 of FA_x_MA_1−x_PbBr_3_ single crystals.

# Section 6 Extraction of deformation potentials

To extract the deformation potential, consistent carrier concentrations generated at different excitation wavelengths must first be ensured. Since the oscillation amplitude is proportional to stress, and stress scales linearly with carrier concentration $(N)$, the oscillation signal at each wavelength was divided by the sample's absorption at that wavelength to normalize the carrier concentration contribution to the stress. Accounting for reflection at the sample surface, the absorption coefficient $A\left( \lambda\right)$ for each wavelength should be expressed as:

$A\left( \lambda\right)=1-R\left( \lambda\right)-T\left( \lambda\right)$ (S2)

where $R\left( \lambda\right)$ is the reflection coefficient of the material for each wavelength, $R\left( \lambda\right)=([{(n-1)}^{2}+k^{2}])/([{(n+1)}^{2}+k^{2}])$,^[18]^ $T\left( \lambda\right)$ is the transmission coefficient at each wavelength, *n* and $k$ are the refractive index and the extinction coefficient, respectively. As the grown crystals are sufficiently thick to absorb all incident photons, $T\left( \lambda\right)$ can be considered negligible. Consequently, the oscillation signal at each excitation wavelength was divided by the corresponding absorption $A\left( \lambda\right)$ (Table S4).

Table S4. Absorption $A\left( \lambda\right)$ of FA_x_MA_1−x_PbBr_3_ single crystals at varied wavelength.

| $A\left( \lambda\right)$  FA Content (x) | At 360 nm excitation | At 400 nm excitation | At 480 nm excitation |
| --- | --- | --- | --- |
| 0 | / | 0.86 | 0.87 |
| 0.4 | / | 0.84 | 0.86 |
| 0.8 | 0.84 | / | 0.86 |
| 1 | 0.83 | / | 0.84 |

Due to the wavelength-dependent penetration depth of light, the energy deposition of the pump light $I(z,t)$ is spatially dependent

$I(z,t)=(1-R)I_{0}f(t)e^{-\alpha z}$ (S3)

where $I_{0}$ is the incident pump intensity, $f(t)$ is the function describes how the pump intensity varies with time, $\alpha$ is the absorption coefficient of the material, and $z$ is the depth into the material from the surface.

Since the induced stress is proportional to the deposited pump energy, we introduce $\eta_{\lambda}$ as the conversion factor between deposited energy and stress, the stress can be then expressed as

$\sigma(z,t)\propto\eta_{\lambda}I(z,t)$ (S4)

Characterizing the frequency differences of the acoustic pulses generated at specific frequencies is essential. Because the coherent acoustic phonons were probed at specific Brillouin frequencies, the measured oscillation amplitude represents only a part of the total acoustic strain. Therefore, to determine the corresponding Brillouin frequencies for each crystal, we need to perform a Fourier transform on the coherent acoustic phonon (CAP) oscillating signal for each crystal. As shown in Figure S4, we obtain the frequency values of 17.9 GHz, 17.8 GHz, 17.2 GHz, and 16.1 GHz for x=0, x=0.4, x=0.8 and x=1 in FA_x_MA_1−x_PbBr_3_ single crystals.


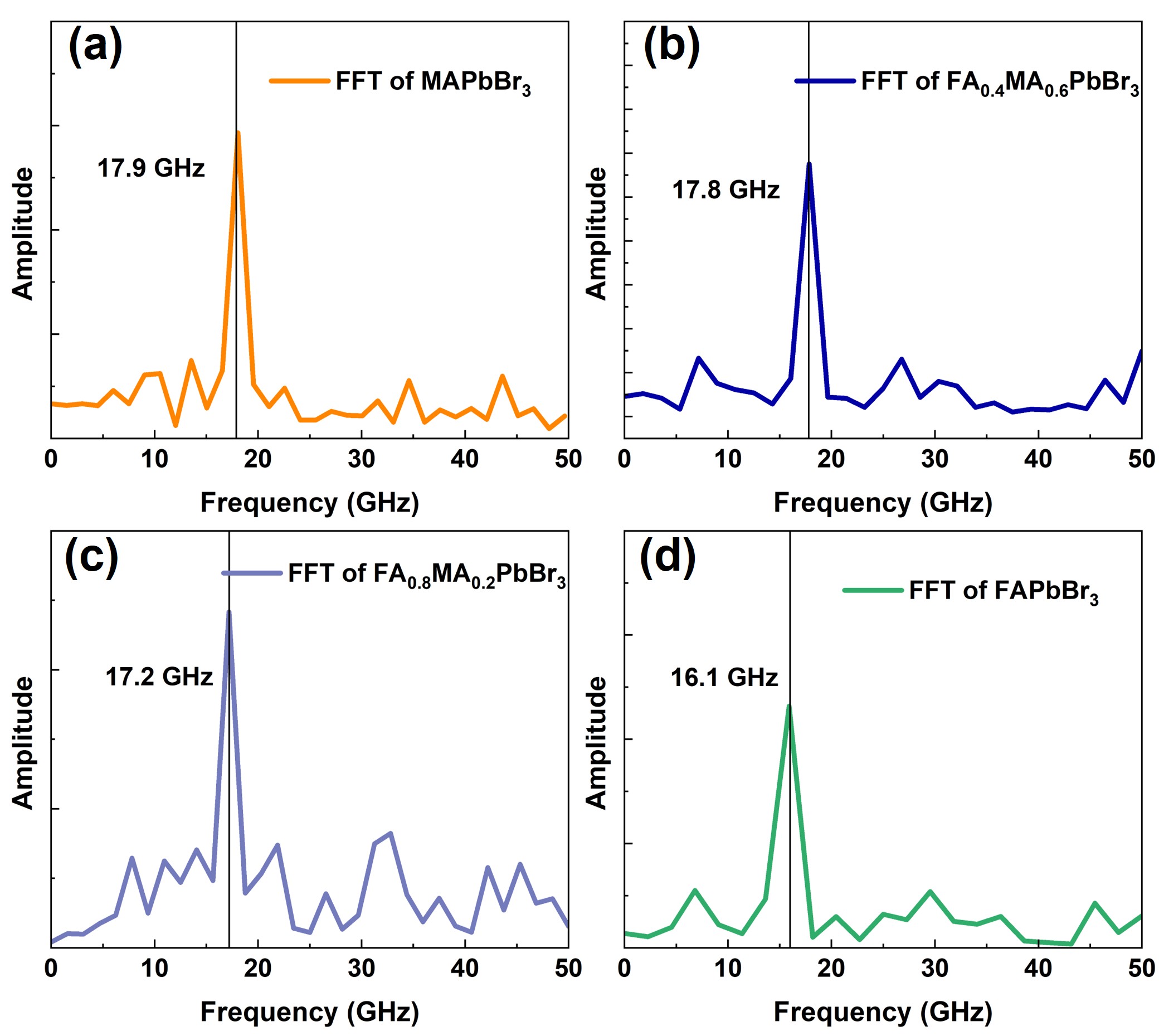


Figure S4. Fourier transform of the oscillation signal for (a) x=0, (b) x= 0.4, (c) x=0.8, and (d) x=1 of FA_x_MA_1−x_PbBr_3_ single crystals.

The spectrum of strain is directly proportional to:^[19]^

$S(\omega)\propto(1-R)\eta_{\lambda}\frac{\omega\tau}{1+\omega^{2}\tau^{2}}$ (S5)

where $\tau=\xi/\nu$ and $\xi=\lambda/{4\pi k}$*.*^[20]^ $\nu$ denotes the speed of sound, and ξ the optical penetration depth in the material.

Using Equation S5, we reproduced the Fourier spectra of the acoustic pulse for pump wavelengths of 400 nm and 480 nm (Figures S5a, b) and for 360 nm and 480 nm (Figures S5c, d), assuming a similar value of $\eta_{\lambda}$. To compare stresses generated at different pump wavelengths via CAP amplitudes at specific frequencies, a correction factor $\gamma$ must be applied. We determined $\gamma$ values of 0.69 (x=0), 0.72 (x=0.4), 0.49 (x=0.8), and 0.51 (x=1). This correction accounts for phonon frequency spectrum changes induced by penetration depth variations.


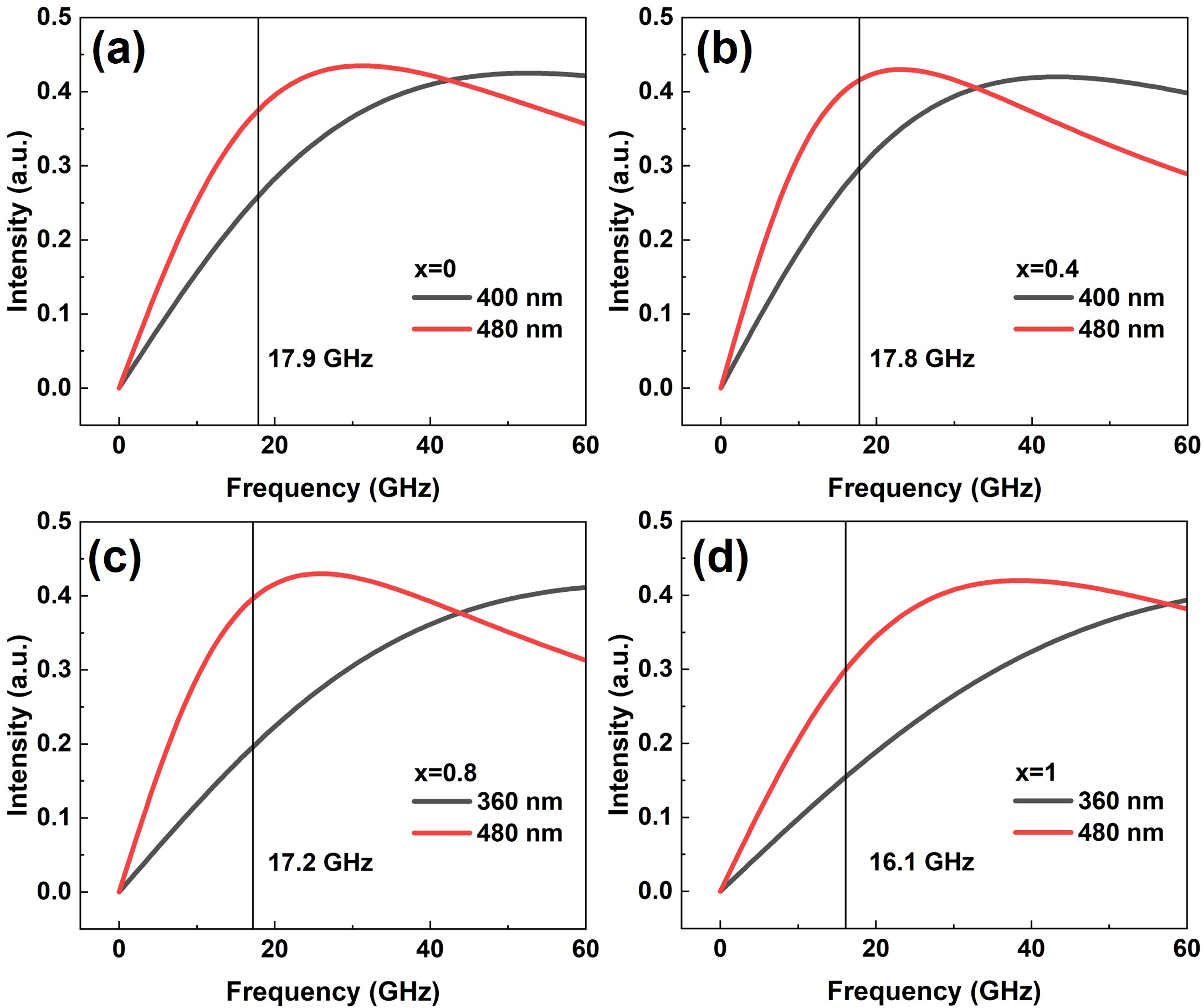


Figure S5. The spectrum of coherent acoustic phonons generated in (a) x=0 and (b) x= 0.4 of FA_x_MA_1−x_PbBr_3_ single crystals for a pump wavelength of either 400 or 480 nm; (c) x=0.8 and (d) x= 1 of FA_x_MA_1−x_PbBr_3_ single crystals for a pump wavelength of either 360 or 480 nm.

As we mentioned in the maintext, combining the thermal expansion stress as well as the deformation potential stress, we can express the total stress of crystals generated by absorbing the pump in different wavelengths in the following form:

$\sigma_{\lambda}=\sigma_{TE,\lambda}+\sigma_{DP, \lambda}=-3B\beta N\frac{{h\nu}_{\lambda}-E_{g}}{C_{p}}-N{(d}_{e}+d_{h})$ (S6)

To extract the deformation potential, the relationship between total stresses generated at different pump wavelengths must be determined. Considering pump wavelengths of 360 nm and 480 nm as examples, we introduce a factor α relating the amplitudes of the CAP oscillation signals. This proportionality factor accounts for the direct relationship between total stress and CAP amplitude, while maintaining consistent deformation potential stress at equal carrier densities across photon energies:

$\sigma_{360}={\alpha\gamma\sigma}_{480}$ (S7)

$\sigma_{TE, 360}-{\alpha\gamma\sigma}_{TE, 480}=(\alpha\gamma-1)\sigma_{DP}$ (S8)

According to Equation S8, we can now calculate the sum of deformation potential ${(d}_{e}+d_{h})$.

# Section 7 Fitting parameters of Brillouin scattering signal under different excitation wavelengths for FA_x_MA_1−x_PbBr_3_ single crystals

Table S5. Fitting parameters of Brillouin scattering signal under different excitation wavelengths for FA_x_MA_1−x_PbBr_3_ single crystals

| FA content  [x] | Probe  [nm] | Pump  [nm] | $t_{c}$  [ps] | $\tau$[ps] | $\tau_{B}$  [ps] | $A_{B}$  [×10^−2^] |
| --- | --- | --- | --- | --- | --- | --- |
| 0 | 700 | 400 | 30 | 56($\pm$0.2) | 990 | 13.0($\pm$0.3) |
|  |  | 480 | 29 | 56($\pm$0.2) | 976 | 7.6($\pm$0.2) |
| 0.4 | 717 | 400 | 37 | 56($\pm$0.3) | 599 | 9.7($\pm$0.2) |
|  |  | 480 | 40 | 57($\pm$0.3) | 596 | 5.7($\pm$0.2) |
| 0.8 | 717 | 360 | 45 | 58($\pm$0.4) | 595 | 6.3($\pm$0.3) |
|  |  | 480 | 30 | 57($\pm$0.4) | 594 | 5.3($\pm$0.2) |
| 1 | 750 | 360 | 55 | 62($\pm$0.2) | 596 | 8.2($\pm$0.3) |
|  |  | 480 | 53 | 63($\pm$0.2) | 591 | 5.4($\pm$0.3) |

# Section 8 Parameters related to FA_x_MA_1−x_PbBr_3_ single crystals deformation potential extraction

Table S6. Parameters related to FA_x_MA_1−x_PbBr_3_ single crystals deformation potential extraction

| FA content  [x] | $B$  [GPa] | $\beta$  [×10^−5^ K^−1^] | $C_{p}$  [×10^6^ J∙m^−3^∙K^−1^] | $E_{g}$  [eV] | $\gamma$ | $\alpha$ | $d_{e}+d_{h}$  [eV] |
| --- | --- | --- | --- | --- | --- | --- | --- |
| 0 | 16.8^[21]^ | 6.7^[22]^ | 1.45^[22]^ | 2.29 | 0.7 | 1.7($\pm$0.05) | 5.69($\pm$1.1) |
| 0.4 | 16.76 | 7.4 | 1.57 | 2.27 | 0.7 | 1.7($\pm$0.04) | 5.76($\pm$1.0) |
| 0.8 | 16.72 | 8.46 | 1.61 | 2.25 | 0.5 | 1.2($\pm$0.07) | −6.52($\pm$0.5) |
| 1 | 16.7^[21]^ | 8.9^[22]^ | 1.69^[22]^ | 2.24 | 0.5 | 1.5($\pm$0.10) | −9.97($\pm$1.8) |

$B$ is the bulk modulus, $\beta$ is the coefficient of linear expansion, $C_{p}$ is the heat capacity, $E_{g}$ is the sample’s bandgap determined from steady-state PL emission peaks (Figure 1c in the maintext), $\gamma$ is the correction factor accounts for the change in the phonon frequency spectrum induced by the variation in penetration depth (see Section 6), $\alpha$ is the ratio between the amplitudes of the oscillation signals induced by pump with different photon energies, and $d_{e}+d_{h}$ is the sum of deformation potentials.

The deformation potential of FA_x_MA_1−x_PbBr_3_ single crystals was calculated using Equation S8 with literature values for the bulk modulus, linear expansion coefficient, and heat capacity. Since these parameters exhibit an approximately linear dependence on composition in mixed perovskites,^[23,24]^ values for compositions lacking reported data (x = 0.8 and 0.4) were estimated by assuming a linear variation with FA content.

# Section 9 Parameters for intrinsic carrier mobility calculation in FA_x_MA_1−x_PbBr_3_ single crystals

Table S7. The parameters for intrinsic carrier mobility calculation in FA_x_MA_1−x_PbBr_3_ single crystals

| FA content  [x] | Type | $m^{*}$ | $d_{e}+d_{h}$  [eV] | $d_{e}-d_{h}$  [eV] | $d$  [eV] | $C$  [GPa] | $\mu$  [cm^2^·V^−1^·s^−1^] |
| --- | --- | --- | --- | --- | --- | --- | --- |
| 0 | e | 0.318 m_0_^[25]^ | 5.69 | −0.75($\pm$0.01) | 2.47($\pm$0.6) | 38.6($\pm$0.2) | 6775($\pm$3300) |
|  | h | 0.305 m_0_^[25]^ |  |  | 3.22($\pm$0.6) |  | 4425($\pm$1670) |
| 0.4 | e | 0.332 m_0_ | 5.76 | −0.75($\pm$0.01) | 2.51($\pm$0.5) | 36.0($\pm$0.4) | 5516($\pm$2120) |
|  | h | 0.346 m_0_ |  |  | 3.25($\pm$0.5) |  | 2954($\pm$980) |
| 0.8 | e | 0.345 m_0_ | −6.52 | −0.75($\pm$0.01) | −3.63($\pm$0.3) | 33.3($\pm$0.5) | 2204($\pm$370) |
|  | h | 0.387 m_0_ |  |  | −2.89($\pm$0.3) |  | 2606($\pm$540) |
| 1 | e | 0.352 m_0_^[26]^ | −9.97 | −0.75($\pm$0.01) | −5.35($\pm$0.9) | 31.8($\pm$0.2) | 923($\pm$310) |
|  | h | 0.408 m_0_^[26]^ |  |  | −4.61($\pm$0.9) |  | 859($\pm$340) |

$m^{*}$ is the effective mass, $d$ is the deformation potential, $C$ is the elastic constant, and $\mu$ is intrinsic carrier mobility calculated at RT (300 K).

The intrinsic mobility $\mu$ of FA_x_MA_1−x_PbBr_3_ was calculated by substituting the effective mass, deformation potential, and elastic constant into the deformation potential theory $\mu=\frac{\left( 8\pi\right)^{1/2}\hbar^{4}eC}{3\left( m^{*} \right)^{5/2}{(k_{B}T)}^{3/2}d^{2}}$. Effective mass values for the x = 0 and x = 1 compositions were taken from the literatures.^[25,26]^ For the intermediate compositions (x = 0.4 and 0.8), where effective mass values were not reported, they were estimated by assuming a linear variation with FA content, supported by the near-linear trend reported for mixed perovskites.^[27]^

# Section 10 Growth conditions of FA_x_MA_1−x_PbBr_3_ single crystals with varied FA content

Table S8. Growth conditions of FA_x_MA_1−x_PbBr_3_ single crystals with varied FA content

| X | Molar ratios | Solution | Concentration [M] | Growth temperature [℃] |
| --- | --- | --- | --- | --- |
| 0 | MABr: PbBr_2_=1:1 | DMF | 0.8 | 60 |
| 0.4 | FABr: MABr: PbBr_2_=1:2:3 | DMF | 0.8 | 60 |
| 0.8 | FABr: MABr: PbBr_2_=2:1:3 | DMF | 0.8 | 60 |
| 1 | FABr: PbBr_2_=1:1 | DMF | 0.8 | 60 |

# REFERENCES

[1] W. Wang, J. Su, L. Zhang, Y. Lei, D. Wang, D. Lu, Y. Bai, *CrystEngComm* 2018, *20*, 1635.

[2] Y. Liu, Y. Zhang, K. Zhao, Z. Yang, J. Feng, X. Zhang, K. Wang, L. Meng, H. Ye, M. Liu, S. Liu, *Adv. Mater.* 2018, *30*, 1707314.

[3] Y. Liu, H. Lu, J. Niu, H. Zhang, S. Lou, C. Gao, Y. Zhan, X. Zhang, Q. Jin, L. Zheng, *AIP Adv*. 2018, *8*, 095108.

[4] N. K. Kumawat, A. Dey, A. Kumar, S. P. Gopinathan, K. L. Narasimhan, D. Kabra, *ACS Photonics* 2015, *2*, 349.

[5] J. N. Fru, N. Nombona, M. Diale, *Physica B*. 2020, *578*, 411884.

[6] B. Wenger, P. K. Nayak, X. Wen, S. V. Kesava, N. K. Noel, H. J. Snaith, *Nat. Commun.* 2017, *8*, 590.

[7] M. I. Saidaminov, V. Adinolfi, R. Comin, A. L. Abdelhady, W. Peng, I. Dursun, M. Yuan, S. Hoogland, E. H. Sargent, O. M. Bakr, *Nat. Commun.* 2015, *6*, 8724.

[8] F. H. Naqvi, J. Ko, T. H. Kim, C. W. Ahn, Y. Hwang, M. Sheraz, S. Kim, *JKPS* 2022, *81*, 230.

[9] K. Tao, C. Xiong, J. Lin, D. Ma, S. Lin, B. Wang, H. Li, *Adv. Electron. Mater.* 2023, *9*, 2201222.

[10] A. A. Zhumekenov, M. I. Saidaminov, M. A. Haque, E. Alarousu, S. P. Sarmah, B. Murali, I. Dursun, X. Miao, A. L. Abdelhady, T. Wu O. F. Mohammed, O. M. Bakr, *ACS Energy Lett.* 2016, *1*, 32.

[11] M. Yao, J. Jiang, D. Xin, Y. Ma, W. Wei, X. Zheng, L. Shen, *Nano Lett.* 2021, *21*, 3947.

[12] S. Chen, O. Nurmikko, *ACS Photonics* 2017, *4*, 2486.

[13] M. C. Brennan, A. Forde, M. Zhukovskyi, A. J. Baublis, Y. V. Morozov, S. Zhang, Z. Zhang, D. S. Kilin, M. Kuno, *J. Phys. Chem. Lett.* 2021, *12*, 8644.

[14] B. M. Sachith, T. Okamoto, S. Ghimire, T. Umeyama, Y. Takano, H. Imahori, V. Biju, *ACS Appl. Energy Mater.* 2025, *8*, 17180.

[15] Y. Liu, B. J. Kim, H. Wu, G. Boschloo, E. M. J. Johansson, *ACS Appl. Energy Mater.* 2021, *4*, 9276.

[16] Z. Wang, C. Shan, C. Liu, X. Tang, D. Luo, H. Tang, Z. Song, J. Wang, Z. Ren, J. Ma, H. Wang, J. Sun, N. Zhang, W. C. H. Choy, Y. Liu, A. K. K. Kyaw, X. W. Sun, D. Wu, K. Wang, *Cell Rep. Phys. Sci.* 2023, *4*, 101363.

[17] G. A. Elbaz, W.-L. Ong, E. A. Doud, P. Kim, D. W. Paley, X. Roy, J. A. Malen, *Nano Lett.* 2017, *17*, 5734.

[18] G. E. Jellison, *Opt. Mater.* 1992, *1*, 151.

[19] P.-A. Mante, C. C. Stoumpos, M. G. Kanatzidis, A. Yartsev, *Nat. Commun.* 2017, *8*, 14398.

[20] Z. Xu, B. Hou, F. Zhao, Z. Cai, H. Shi, Y. Liu, C. L. Hill, D. G. Musaev, M. Mecklenburg, S. B. Cronin, T. Lian, *Nano Lett.* 2021, *21*, 8017.

[21] A. C. Ferreira, A. Létoublon, S. Paofai, S. Raymond, C. Ecolivet, B. Rufflé, S. Cordier, C. Katan, M. I. Saidaminov, A. A. Zhumekenov, O. M. Bakr, J. Even, P. Bourges, *Phys. Rev. Lett.* 2018, *121*, 085502.

[22] T. Haeger, R. Heiderhoff, T. Riedl, *J. Mater. Chem. C* 2020, *8*, 14289.

[23] U.-G. Jong, C.-J. Yu, Y.-M. Jang, G.-C. Ri, S.-N. Hong, Y.-H. Pae, *J. Power Sources* 2017, *350*, 65.

[24] M. Šimėnas, S. Balčiu̅nas, Š. Svirskas, M. Kinka, M. Ptak, V. Kalendra, A. Ga̧gor, D. Szewczyk, A. Sieradzki, R. Grigalaitis, A. Walsh, M. Ma̧czka, J. Banys, *Chem. Mater.* 2021, *33*, 5926.

[25] M. Borges-Martínez, M. Saavedra-Torres, E. Schott, X. Zarate, *Mater. Today Commun.* 2023, *34*, 105324.

[26] Z. Muhammad, P. Liu, R. Ahmad, S. Jalali Asadabadi, C. Franchini, I. Ahmad, *Phys. Chem. Chem. Phys.* 2020, *22*, 11943.

[27] U.-H. Ko, J.-H. Ri, J.-H. Jang, C.-H. Ri, U.-G. Jong, C.-J. Yu, *RSC Adv.* 2022, *12*, 9755.
